# Design and develop a prototype fixed column using bacterial cellulose and ferric chloride for Cr(VI) removal

**DOI:** 10.3389/fsysb.2026.1844304

**Published:** 2026-07-01

**Authors:** Uriel Fernando Carreño Sayago, Vladimir Ballesteros Ballesteros, Angelica Maria Lozano Aguilar, Jorge Luis Nisperuza

**Affiliations:** Investigador de Fundación Universitaria los Libertadores, Bogotá, Colombia

**Keywords:** adsorption, biomass, cellulose bacterial, Ferric chloride, remotion

## Abstract

Aquatic ecosystems worldwide are experiencing a decline in their ecosystem services, primarily attributable to the irresponsible discharge of heavy metals, including chromium (VI) in some cases. This heavy metal bioaccumulates, harming the survival of aquatic organisms essential to human life. One potential solution to this problem is to treat effluents containing these heavy metals before they reach rivers and wetlands. An effective and easily implemented treatment method involves the use of fixed-column continuous-flow systems. Fixed columns can be constructed using biomass that adsorbs heavy metals, such as bacterial cellulose, a polysaccharide with 100% purity. Furthermore, the adsorption capacity of the material could be enhanced by using Ferric chloride, while EDTA has been shown to prolong the duration of adsorption cycles. Therefore, the objective was to design and develop a prototype fixed column using bacterial cellulose and Ferric chloride for Cr(VI) removal. Various Cr(VI) adsorption processes were analyzed, and a pilot system was developed using bacterial cellulose and three different concentrations of Ferric chloride. After six adsorption cycles, it was determined that the ideal treatment mixture would have a concentration of 85% bacterial cellulose and 15% Ferric chloride, achieving an adsorption capacity of 456 mg/g. This treatment system would provide a practical solution for reducing the impacts of Cr(VI) on aquatic ecosystems.

## Graphical Abstract

**Figure:**
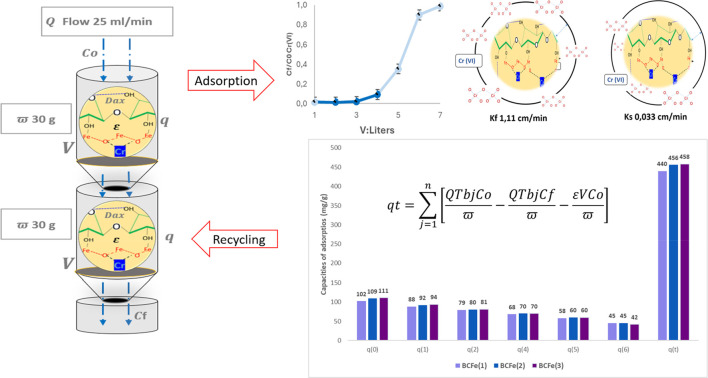

## 1 Introduction

In accordance with Sustainable Development Goal six, which stipulates the necessity of “Ensure availability and sustainable management of water and sanitation for all,” this objective has not been realized in various regions of the world, notably Latin America. This is primarily attributable to the negligence of diverse industrial sectors that have been responsible for the discharge of heavy metals into sewage systems, subsequently resulting in the contamination of wetlands, rivers, and lagoons. The impacts caused by these heavy metals are frequently irreversible, resulting in a diminution of the ecosystem services pro-vided by different bodies of water. One heavy metal that merits particular attention due to its extensive industrial application is Cr(VI), a compound that is employed extensively in the tanning, metal, and paint industries (Sable et al., 2024; Saud et al., 2022; Irshad et al., 2023; Mohanty et al., 2023; Oladimeji et al., 2024). Effluent concentrations in tanneries have been found to exceed 1,000 mg/L of Cr (VI) (Camacllanqui and Huamanlazo, 2025; Xie, 2024; Wang et al., 2023). This heavy metal bioaccumulates in macroinvertebrates, plants, and, especially, fish. It therefore poses a significant threat to human health (Nnaji et al., 2023; Jamil et al., 2023). Consequently, it is imperative to formulate cost-effective strategies for the sanitation of these bodies of water. One such technique is the chemisorption process, which utilizes adsorbent biomasses (Georgin et al., 2023; Zhao et al., 2024). Currently, various research centers worldwide are focused on designing new ways to remove heavy metals present in industrial effluents, and one such new approach involves adsorbent biomass (Hamadneh et al., 2026; Emenike et al., 2026; Gürkan et al., 2023). This biomass has the intrinsic capacity to chemisorb heavy metals by accumulating them in its multiple active sites (Hoang et al., 2022; Gilkes et al., 1992). Bacterial cellulose (BC) is a polymer produced by bacteria, primarily from the genera *Komagataeibacter, Acetobacter, Sarcina ventriculi, and Agrobacterium.* Through this culture medium, kombucha is produced via industrial fermentation processes, supplemented with yeast, tea, and sugar (Sayago et al., 2024; Li et al., 2024; Sayago et al., 2023; Silva et al., 2021). The biomass under scrutiny is characterized by a cellulose composition of 100% (Oktaviani et al., 2023; Leal et al., 2021). These properties position it as a potentially viable biomass for the construction of treatment systems aimed at the removal of heavy metals from contaminated effluents. This is due to the oxidation of the biomass, increasing its active sites (Amar et al., 2024). One of the objectives of these experiments, which are based on continuous processes and adsorbent biomasses, is to understand the scaling process. This scaling can be achieved through the utilization of mathematical models of material balances, which subsequently give rise to the development of external film models, designated as K_f_, and internal film models, denoted as K_s_ (Kavand et al., 2017; Padma et al., 2025; Wei et al., 2025). These parameters are used to determine the area of influence and the amount of biomass along with the design volume, adsorption capacities, and design flow rates (Chepkwony et al., 2025; Mahmoud et al., 2025). It is evident that these models facilitate the adjustment of design and development parameters of treatment processes on a larger scale. Such parameters include, but are not limited to, flow rates, inlet concentrations, biomass densities, the amount of biomass to be used, surface area, and volume of water to be treated (Stefan et al., 2023; Zhang L. et al., 2025; Simonin and Bouté, 2016). Another pivotal design parameter pertains to the elution and reuse process, incorporating various cycles of this process, which has been demonstrated to enhance adsorption capacities considerably, thereby rendering the process highly viable for the construction of a functional treatment system (Alsaeedi et al., 2023; Sayago and Ballesteros, 2024). EDTA has been identified as the most effective reagent employed in elution processes to enhance adsorption capacities, thereby facilitating the reuse of biomasses for more than six treatment cycles (Jiang et al., 2023; Zhang Y. et al., 2025). Currently, many biomass studies using Ferric chloride are being conducted, but these are primarily at laboratory scales, while rivers, lakes, and wetlands continue to be contaminated with Cr(VI), such as the Bogotá River. This is mainly due to the lack of design and implementation of economical, reliable, and easy-to-install treatment systems. Therefore, a project was initiated to Design and develop a prototype fixed column using bacterial cellulose and ferrous chloride for Cr(VI) removal.

## 2 Methods and materials

### 2.1 Bacterial cellulose production

The production process was carried out at the research facilities of Universidad Libertadores. The bioreactors were designed and built for the continuous generation of bacterial cellulose. They have a capacity of 2 L. The culture medium is prepared with 1.5 L of distilled water, followed by the addition of red tea (4 g) and sugar (5 g). The medium is heated to 80 °C to ensure initial sterilization and then allowed to cool. Subsequently, 330 ml of SCOBY (symbiotic culture of bacteria and yeast) are added.

The initial pH is neutral. Magnetic stirring is not used; it is a batch process. pH, temperature, and dissolved oxygen levels are monitored daily. The biofilm is allowed to grow for approximately 2 weeks, washed with copious amounts of water, and then subjected to an air-drying process. After drying, the material is ground to a diameter of 0.211 mm using a blade mill and sieved through a 70 mesh sieve (US standard sieve).

### 2.2 Adsorption experiments

Cr(VI) Stock Solution: A 1,000 mg/L solution was prepared with distilled water and potassium dichromate (K_2_Cr_2_O_7_). This stock solution was used to prepare Cr(VI) test solutions of 400, 600, and 1,000 mg/L. The batch adsorption study was performed at 24 °C. Samples were taken from each 500 mL treated volume interval and analyzed to determine the residual chromium concentration. Samples of 100 microliters were obtained and analyzed using the diphenylcarbazide method.

For this purpose, 900 microliters of phosphate buffer were prepared, adjusted to a pHof2 and a purity of 90% (H_3_PO_4_). The diphenylcarbazide solution is composed of diphenylcarbazide (1,5-diphenylcarbazide) and the solvent acetone, with a concentration of 0.5% (97% purity). A 200-μL solution of diphenylcarbazide solution was prepared using 120 microliters of acetone (97% purity, w/v) and 80 microliters of diphenylcarbazide. This solution was then combined with 100 microliters of residual chromium sample and transferred to an Eppendorf tube. Subsequently, the solution was transferred to an adsorption cell, where the absorbance was measured at 540 nm. The measurement uncertainty of this study indicates that measurements of heavy elements, specifically Cr(VI), can be performed with an uncertainty level of approximately 4%, according to APHA (American Public Health Association) Standard Methods for Water and Wastewater Analysis.

### 2.3 Chromium measurement

Chromium content was measured using a spectrophotometer (Evolution 300) by monitoring changes in light adsorption. All procedures for the determination of chromium in water and substrates were carried out following APHA (American Public Health Association) procedures for standard analysis (Standard Methods for the Analysis of Water and Wastewater).

### 2.4 Obtaining ferric -modified cellulose

Materials containing Ferric chloride (Fe_3_ + [FeCl_3_ · 6H_2_O]) (Danish et al., 2022). Were mixed with bacterial cellulose to impregnate the reagent into the biomass by dry weight.

The mixture was homogenized during the process, where the pilot-scale system contained 60 g of the composite material, thus the combination was:

- 95% (w/w) bacterial cellulose (57 g) and 5% Ferric (III) chloride (3 g). BCFe (1).
- 85% (w/w) bacterial cellulose (51 g) and 15% Ferric (III) chloride (9 g). BCFe (2).
- 75% (w/w) bacterial cellulose (45 g) and 25% Ferric (III) chloride (15 g). BCFe (3).

### 2.5 Experiments conducted within a column

**FIGURE 1:**
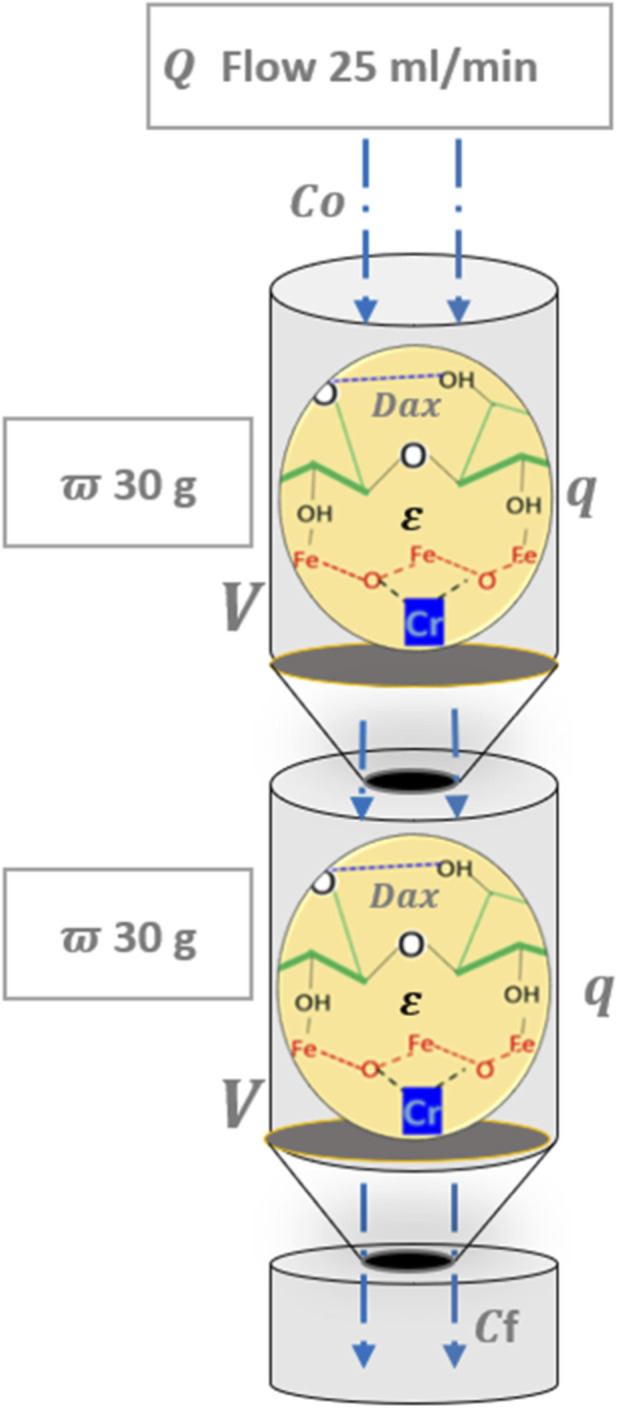
Treatment design, with two compartments, each containing 30 g of Ferric -modified cellulose. With a flow rate of 25 ml/min.

The system was constructed with two layers of cellulosic biomass, each weighing 30 g and measuring 4.5 cm in diameter, secured with ferric chloride. The columns exhibit a funnel effect at the end of the treatment process, increasing contact between the biomass and the fluid. Designing a system with more compartments helps to mitigate vortex effects (Zhang Y. et al., 2025; Ojembarrena et al., 2023). The flow rate was set at 25 ml/min, regulated at the inlet and secured by dripping. The biomass density was kept constant, as were the temperature (20 °C) and pressure (1 bar). An additional compartment was designated for final sampling. Tests were performed under neutral pH conditions, favorable for the adsorption process in this type of biomass and similar to those of industrial effluents. The chromium (VI) concentrations evaluated in this study ranged from 600 to 300 mg/L. For statistical analysis, two tests were performed for each treatment, and the average of the data obtained was calculated, where Student’s *t*-distribution was used. These results were analysed using this method due to their representativeness in previous studies on heavy-metal-adsorbing biomasses. In Figure 1. Show the system of treatment.

### 2.6 The desorption and subsequent adsorption process

Elutions and reuse. Using the reagent EDTA Na_2_ (97% purity), elutions were performed when the biomass was saturated with chromium. Similar to the treatment, the solution was passed through the system in fixed columns, with an EDTA concentration of 500 mg/L, approximately 1 L of this solution. To remove excess EDTA, it was washed with 1 L of distilled water.

### 2.7 Mathematical modelling

Equation 1 representing the balance material of system, with all the parameters essential of the design of one process of treatment with biomass adsorbent in the column. In pilot-scale treatment processes, such as the amount of biomass (ϖ), porosity (ε), initial contaminant concentration (C_o_), output concentration (C_f_), adsorption capacity mg/g (q), input flow rate (Q) ml/min, and the volume (V) to be treated, is Equation 1.

$$\varepsilon \left(*\right)\frac{\mathrm{Co}}{\partial t}+V\frac{q}{\partial t}+Q\frac{\mathrm{Cf}}{\partial t}‐\mathrm{Dax},\varepsilon \;\frac{\partial C}{\partial Z}$$ (1)

The axial dispersion coefficient is one that involves the density of the particle and the porosity, Equation 2.

$$Dax=\frac{1.8\;\text{QDp}}{\varepsilon }$$ (2)

*D*_*ax*_-Axial dispersion coefficient (m_2_/h), D_p_-Particle density (kg/m)

The porosity (ε) is obtained through Equation 3.

$$\varepsilon =1‐\frac{\mathrm{df}}{\mathrm{Dp}}$$ (3)

D_p_-Particle density (kg/m), D_f_-Density of the biomass (g/ml)

The density ratio is an ideal design parameter for scaling up treatment systems. It is the ratio of the microparticle density to the total biomass. If the biomass density is much higher than the microparticle density, it is essential that the particle density be slightly higher than the biomass density, in the range of 60%–90%. This indicates that the values ​​of this design parameter should be between 0.4 and 0.75 (Ojembarrena et al., 2023; Worch, 2012). In the volume (V) is obtenided with the Equation 4.

$$v=\frac{\varpi *q+\frac{\varepsilon \;Co}{Df}\;}{Cf}$$ (4)

$\varpi \;‐\text{Amount of biomass used }\left(g\right),T‐\text{Breakthrough }\left(\min\right)$

This equation could be used to design treatment systems, as it takes into account the parameters for building scaled systems. This equation is ideal when biomass reuse processes are applied via elution processes, where adsorption capacities following different elution processes are considered (Sayago, 2021). The Equation 5 is:

$$\mathrm{qt}=\sum_{j=1}^{n}\left[\frac{\mathrm{QTbjCo}}{\varpi }‐\frac{\mathrm{QTbjCf}}{\varpi }‐\frac{\varepsilon \mathrm{VCo}}{\varpi }\right]$$ (5)

Where: Q = design flow (ml/min), T_bj_ - Break time of use number j (min), C_f_ - End Cr(VI) (mg/ml); L Co = initial Cr(VI) (mg/ml), V-Volume of the system (ml), ε-Porosity, ϖ -amount of biomass used (g); q_T- Total adsorption capacity (mg/g).

Extra particle diffusion is a model for predicting the amount of heavy material being adsorbed to the top of the biomass particle, the Equation 6 is:

$$\varepsilon \frac{\partial Co}{\partial t}+V\frac{\partial q}{\partial t}=\frac{Kf}{L}\left(C-Cs\right);$$ (6)

In which it can be represented through the graph, together with the initial and final concentrations, vs. the treated volume.

$$\mathrm{Ln}\;\frac{Cr\left(VI\right)\;}{Cs}=\frac{kf}{L}t$$ (7)

K_f_--Mass transfer coefficient in the liquid particle (m/h), L- Length, relations volume and area, Cs - Equilibrium concentration of pollutant (Sayago, 2021).

Intraparticle diffusion K_s_ is when there are chemisorption processes and they are vital in modelling the behaviour of adsorption isotherms, due to processes within the biomass particle (Danish et al., 2022). The Equation 8 is:

$$\frac{\partial q}{\partial t}=\text{DpKs}\left(\text{qs}-q\right)$$ (8)

Due to the influence of the isotherm, the K_s_ coefficient could be adjusted through the isotherm equations of Langmuir and Freundlich. The Equation 9 is:

$$\text{ksDp}\left(\text{qs}-q\right)=-\frac{\text{Kf}}{L}\left(c-\text{cs}\right)$$ (9)

If it is the Freundlich Equation 10:

$$\text{qs}=K{\left(\text{Cs}\right)}^{n}$$ (10)

The resulting expression with Freundlich modeling, Equation 11.

$$\text{ks Dp Ks}{\left(\text{Cs}\right)}^{n}-\text{Ks Dp }q=-\frac{\text{Kf}}{L}\left(C-\text{Cs}\right)$$ (11)

Equation 11 remains to obtain the intraparticle diffusion constant when adjusted to this Freundlich isotherm

$$\text{Ks}=\frac{\text{Kf}\left(C-\text{Cs}\right)}{\text{pp}\left(\text{KCs}{Cs}^{n}-q\right)L}$$ (12)

The intraparticle coefficient can be determined by utilizing Equation 12 in the event that the adsorption process is adjusted to the Freundlich isotherm. The determination of thermodynamics is pivotal in the context of multilayer processes and heterogeneous processes, such as those occurring in zeolites and chemical chelating agents, which are characterised by their high porosity. The validity of the resultant Equation 13 is contingent upon the temporal adjustment of the process to the Langmuir isotherm.

$$q_{S}=\frac{\text{qmKlCs}}{1+\text{BCs}}$$ (13)

Where Kl is the Langmuir adsorption constant, with this parameter thermodynamic processes could be adjusted, the Equation 14 is:

$$\text{ks}\left(\text{qs}-q\right)=k_{F}\frac{As}{V}\;t$$ (14)

Clearing from Equation 7, the constant is Ks.

$$K_{S}=\frac{\text{KfCs}}{\text{pp}\left(\text{qmB}-\text{qb}-q\right)}+\frac{\text{AsKfCs}}{\text{Vpp}\left(\text{qmB}-\text{qb}-q\right)}$$ (15)

Equation 15 facilitates the calculation of the intraparticle coefficient when the adsorption process is adjusted to the Langmuir isotherm, a characteristic of natural adsorbent biomasses from biological processes. It is evident that this isotherm is capable of determining both monolayer and homogeneous processes. Furthermore, it has been demonstrated that these processes can be adjusted to predictable, deterministic adsorption processes.

## 3 Result

### 3.1 Fourier-transform infrared spectroscopy (FTIR)

**FIGURE 2:**
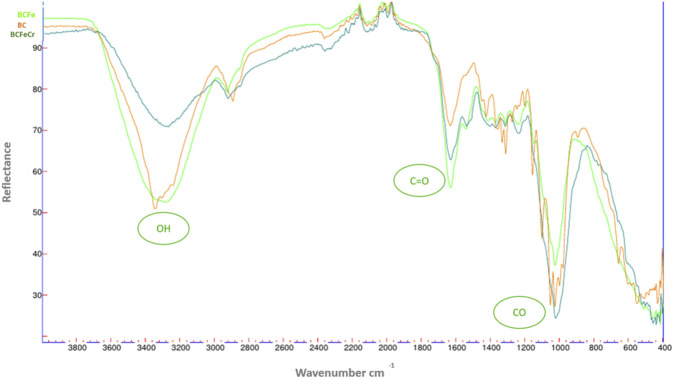
Analysed of FTIR.

As illustrated in Figure 2, the addition of ferric chloride to bacterial cellulose results in the characteristic spectra of the material. Following this, the adsorption of Cr (VI) into the biochemical structure of the cellulose is evident. The hydroxyl (OH) groups present within the 3,400 cm^-1^ band are instrumental in the process of removing Cr (VI) ions.

Figure 2 shows shifts in wave number and differences in intensity for the characteristic functional groups in the three biomasses evaluated: BC, BCFe, and BCFeCr. Upon contact with cellulose, Ferric Chloride undergoes an oxidation process that leads to the formation of FeOOH complexes, reflected in the stretching of the (OH) band to 3340 cm^-1^, characteristic of cellulosic biomass, which is accentuated by this reagent. Furthermore, the peaks corresponding to the carbonyl groups (C=O and CO) also showed an alteration in response to the incorporation of ferric iron into cellulose. This transformation of the biomass favors the cation exchange process between these functional groups and the heavy metal Cr(VI) (Xu et al., 2022). Following the adsorption process, comparison of the spectra of the three cellulose samples reveals a decrease in the intensity of the characteristic peaks (OH, C=O, and CO) of the biomass after the incorporation of Cr(VI). This observation indicates a chemical interaction of the biomass, suggesting that the Cr(VI) reacted biochemically with these groups. The most notable of these is the (OH) band, attributable to the coordination between this group and the Cr(VI) metal ion, demonstrating that it is the most significant in the adsorption process of heavy metals by adsorbent biomasses (Sayago et al., 2025). Compared to the biomasses, the one showing the greatest change in intensity of the (OH) group is the BCFeCr biomass, indicating that the cellulose biomass removed this heavy metal through ion exchange (Wang et al., 2018).

### 3.2 SEM and EDS analysis

**FIGURE 3:**
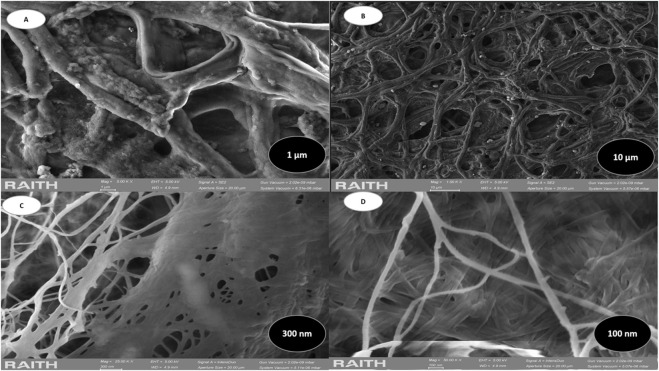
BC analysis. **(A)** Shows SEM morphology at 1 µm. **(B)** Shows SEM morphology at 10 µm. **(C)** Shows SEM morphology at 300 nm. **(D)** Shows SEM morphology at 100 nm.

Figure 3 shows some characteristic images of the bacterial cellulose used in this research, with representations obtained through SEM analysis.

Bacterial cellulose, having 100% purity, that is, contains an ideal totality of this polysaccharide, making it homogeneous and uniform (Chen et al., 2020). It is a pure polysaccharide, without lignin and hemicellulose, making it favorable to the adsorption processes of heavy metals. It can see in the different figures (from 3a to 3D) that three-dimensional nanoporous networks of cellulose nanofibers are formed (Yingkong and Tanskul, 2019). Also, an EDS mapping was performed, which revealed a distribution of the elements Oxygen (O), Carbon (C), Iron (Fe) and Chromium (Cr) throughout its biochemical complex. The collection of the elements shown in Figure 3D, are summarized in Table 1.

**TABLE 1:** Physicochemical characterization of the BCFe sample.

Elements	Weight	%
Carbon (C)	36.6	41.67
Oxygen (O)	30.3	38.94
Iron (Fe)	12.13	11.5
Chromium (Cr)	11.2	10.0

The distribution of elements within the biomass loaded with Iron and Cr(VI) is evident, thereby confirming a removal process from the bacterial cellulose. The homogeneous distribution of these elements renders the process more reliable than that of heterogeneous biomass.

### 3.3 Analysis of Cr(VI) removals

**FIGURE 4:**
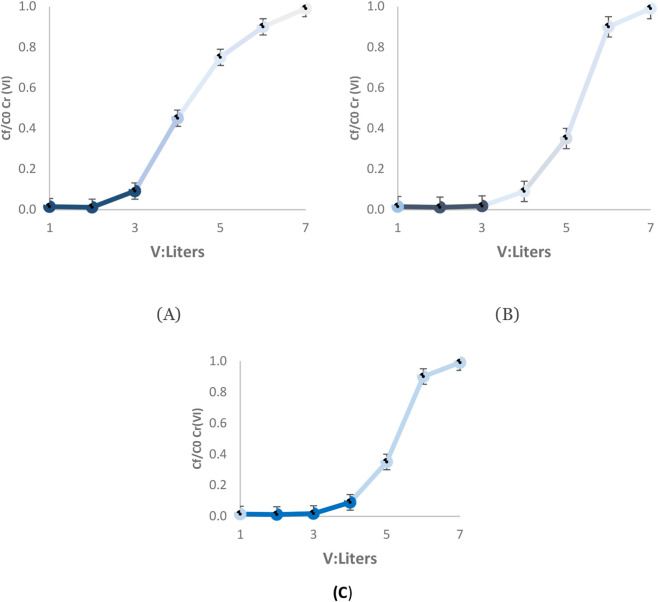
Treatment with different biomass. **(A)** Treatment with the biomass BCFe (1). **(B)** Treatment with the biomass BCFe (2). **(C)** Treatment with the biomass BCFe (3).

Figure 4 show the Cr (VI) removal processes by biomasses. The results of the Cr(VI) removal process by biomass were performed in duplicate. The graphs represent the average of the two data points, and the error bars represent the standardized errors.

The study demonstrated the continuous treatment system’s and pilot scale’s remarkable adsorption capacity, leading to the effective removal of Cr (VI). In accordance with predictions, cellulose treated with a lower portion of ferric chloride exhibited a reduced capacity to treat litres of water in comparison to the other two concentrations. However, the treatment obtained was nonetheless significant, with approximately 3 L of water contaminated with Cr (VI) being treated, resulting in the removal of around 99% of this metal in the final concentration. The remaining two treatment systems yielded analogous outcomes. The BCFe (2) treatment system treated approximately 3.5 L, while the BCFe (3) system removed a mere 300 ml more, resulting in a total of 3.8 L. When chromium in the form of dichromate Cr_2_O_7_ comes into contact with biomass oxidized with ferric chloride, reactions occur with the (H^+^) of the biomass and the oxygens of the chromium structure, reducing it to chromium oxide Cr_2_O_2_, Cr (III) and subsequently chemiadsorbing into the same biomass (Sayago et al., 2025).

### 3.4 Process of the modelations

The process of modelations is the values of the main parameters used to describe the adsorption in Table 2.

**TABLE 2:** The values of the main parameters used to describe the adsorption.

Parametric	BCFe (1)	BCFe (2)	BCFe (3)
D_ax_	1.12 x 10^−3^	1.14 x 10^−3^	1.2 x 10^−3^
q_m_ (mg/g)	102	109	111
T_b_ (min)	120	160	168
K_f_ (cm/min)	1.12	1.18	1.21
Adjustment Langmuir (R^2^)	0.99	0.99	0.98
Adjustment Freundlich (R^2^)	0.93	0.92	0.95
K_s_ (cm/min) Freundlich	0.031	0.033	0.034
K_s_ (cm/min) Langmuir	0.032	0.033	0.035

Utilizing Equation 2, the axial dispersions of the various biomasses were derived, exhibiting analogous trends across the three assessments, attributable to the quantity of heavy metal employed. As demonstrated in Figure 2, the adsorption capacities and breakthrough times were obtained. In this study, the adsorption capacities were corroborated with this parameter. The breakthrough times were found to be contingent upon the flow rate reported in the pilot system (25 ml/min). For instance, the BCFe (1) biomass obtained following treatment with 3,000 ml of treated water yielded a result of 120 min of breakthrough. The BCFe (2) and BCFe (3) biomasses, which exhibited comparable yields, also attained substantial values of this parameter, at 160 and 168 min, respectively. It was found that an adjustment could be made through the use of isotherm evaluation processes and equilibrium concentrations. The Langmuir isotherm was selected for this adjustment, as it is considered to be ideal for adjusting and modelling this type of biochemical material, with R^2^ = 0.99 and Freundlich R^2^ = 0.93. This is due to the structural homogeneity of the material, which is a biomass with a monolayer and 100% bacterial cellulose. This makes it predictable when obtaining relevant information about its adsorption capacities and contaminant distributions (Min and Zhang, 2014; Dassekpo et al., 2025). The adsorption rate constants for each biomass were obtained through Equation 7. It has been demonstrated that bacterial cellulose biomasses with different concentrations of ferric chloride exhibit interesting adsorption rates. For instance, the BCFe (1) biomass has a rate of 1.12 cm/min, the BCFe (2) biomass reaches 1.18 cm/min, and the BCFe (3) biomass has a rate of 1.21 cm/min. This is achieved by relating the desired treatment volume to the surface area and breakthrough times and concatenating the inlet and outlet concentrations. Moreover, the K_f_ constant is well-suited to the design of treatment systems with fixed biomasses in a column with a descending fluid. This is due to the fact that the inlet loads can be modelled along with the amount of biomass and volumes to be treated (Tuomikoski et al., 2021).

**FIGURE 5:**
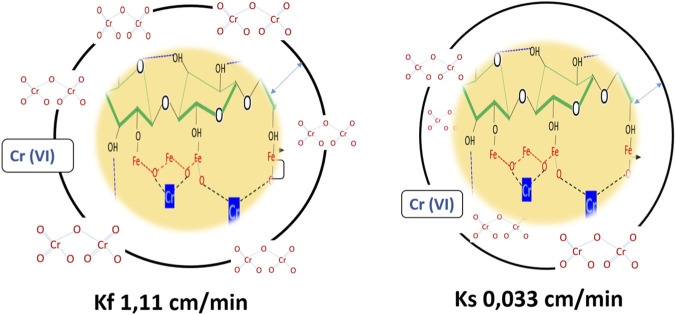
Extraparticle diffusion and Intraparticle Diffusion of particle of cellulose bacterial with Iron.

The intraparticle adsorption constant, K_s_, was determined by utilizing Equation 15 in conjunction with a representative fit of the Langmuir isotherm. Utilizing the aforementioned equation, the adsorption capacity constant, K_s_, was determined to be 0.035, 0.037 and 0.038 (cm/min) for the biomasses BCFe (1), BCFe (2) and BCFe (3), respectively. It is posited that, by employing this model, it is possible to determine the intraparticle chemisorption rate of the heavy metal to the evaluated biomasses. The utilization of the aforementioned equation facilitates the design of treatment systems. This is achieved through the modelling of the densities, biomasses to be employed, and, most significantly, the volume to be treated. The densities of the biomass and microparticles in question are of fundamental importance. In the modelling processes of fixed column treatment systems, it has been found that a diameter of less than 0.212 mm is ideal due to the direct relationship between the biomass and the contaminant, favouring chemisorption (Emenike et al., 2026; Wang and Guo, 2023; Wang and Guo, 2023). The Figure 5 show the analysed of one intraparticle and extra particle of cellulose bacterial with Iron.

### 3.5 Desorption-elution and reuse

**FIGURE 6:**
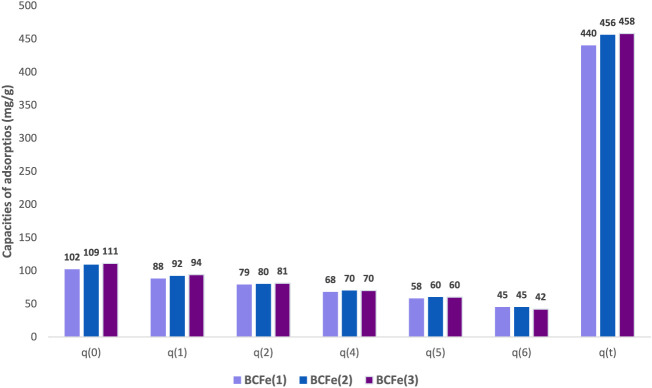
Adsorption capacities in biomass in each treatment cycle.

In research processes using biomass adsorbents, elution and subsequent reuse are essential for process design due to the increased adsorption capacity. In Figure 6 show the adsorption capacities in the different adsorption processes biomasses.

All biomasses utilized in the heavy metal removal process yielded encouraging outcomes, exhibiting an increase in the initial adsorption capacity by almost 4.5 times. Summing all these adsorption capacities gives a cumulative total of more 440 mg/g, demonstrating that this process is of vital importance, increasing the economic and environ mental sustainability. This action has been demonstrated to enhance the technical viability and economic efficiency of this large-scale treatment system. The enhancement of adsorption capacities has been demonstrated in research conducted with adsorbent biomasses (Russo et al., 2015; González et al., 2023; Wiśniewska et al., 2022), wherein this recycling process is imperative in treatment processes. The BCFe (2) biomass demonstrated optimal performance, attaining a capacity of 456 mg/g, though this was marginally lower than that of the BCFe (3) biomass. However, the BCFe (2) biomass exhibited a reduced quantity of ferric chloride. In order to minimise the use of this reagent and consequently reduce the cost of the system, it is recommended that BCFe (1) be used for treatment with a reduced pollutant load.

Subsequently, some comparisons were made with other relevant investigations, observing the adsorption capacity, the chemical elucidating agent and the sum of the adsorption capacity; Table 3 shows summaries of some investigations.

**TABLE 3:** References about biomass adsorbent and capacities of adsorptions.

Reference	Biomass	Heavy metal	Elutionary chemical	Capacity (mg/g)	Capacity (mg/g) in different cycles
Present	BCFe (1)	Cr (VI)	EDTA	111	458
González et al. (2023)	Activated Biocarbons	Cd (II)	​	240	​
González et al. (2023)	Activated Biocarbons	Pb(II)	​	340	​
Wiśniewska et al. (2022)	Biomass and biochar-based adsorbents	Cu(II)	​	177	​
Wiśniewska et al. (2022)	Biomass and biochar-based adsorbents	Pb(II)	​	178	​
Rohman et al. (2024)	Chlorella pyrenoidosa algal biomass	Pb(II)	​	159	​
Sireesha et al. (2022)	Biomass and biochar-based adsorbents (Delonix leaves (Gulmohar)	Ni	H_2_SO_4_	33	150
Li et al. (2022a)	N-doped Medulla tetrapanacis biochar	Cu (II)	HCl	468	980
Emenike et al. (2026)	Carbon active	Cr (VI)	HNO_3_	36	74
Li et al. (2022b)	MgO-modified rice husk biochar	Cu(II)	NaOH	166	600
Li et al. (2022b)	MgO-modified rice husk biochar	Cr(VI)	NaOH	47	140
Licona et al. (2022)	Sugarcane bagasse and orange peel to obtain carbon foams	Pb(II)	NaOH	450	968
Adegoke et al. (2022)	Acid-activated Posidonia oceanica	Pb(II)	H_3_PO_4_	240	631
Zhang et al. (2022)	Alginate-based hydrogel	Cr(VI)	NaOH	86	151
Zhang et al. (2022)	Alginate-based hydrogel	Pb(II)	NaOH	139	420
Zhang et al. (2022)	Alginate-biomass hydrogel	Cd(II)	HCl	110	380

After adsorption and elution processes, adsorption capacities increase. For example, biomass (Licona et al., 2022) has excellent adsorption capacities, and the sum of these capacities reaches 980 mg/g of Pb (II). Similarly, modified biomass (Zhang et al., 2022) achieved a sum of 600 mg/g of Cu (II) removal. Cr (VI) removals in biomasses (Adegoke et al., 2022; Zhang et al., 2022) did not exceed 160 mg/g, because this heavy metal is one of the most difficult to remove in contaminated environments.

### 3.6 Isotherms after the elution process.

The parameters established above were analysed after treatment cycle six to determine the conditions under which the process ended. In the Table 4 show the values of the main parameters used to describe the adsorption.

**TABLE 4:** The values of the main parameters used to describe the adsorption in the cycle six.

Parametric	BCFe (1)	BCFe (2)	BCFe (3)
D_ax_	1.0 x 10^−3^	1.0 x 10^−3^	1.1 x 10^−3^
q_m_ (mg/g)	45	45	42
Tb (min)	60	60	59
K_f_ (cm/min)	0.35	0.33	0.32
Langmuir (R^2^)	0.91	0.92	0.92
Freundlich (R^2^)	0.95	0.94	0.95
K_s_ (cm/min) Freundlich	0.022	0.025	0.021
K_s_ (cm/min) Langmuir	0.021	0.021	0.022

**FIGURE 7:**
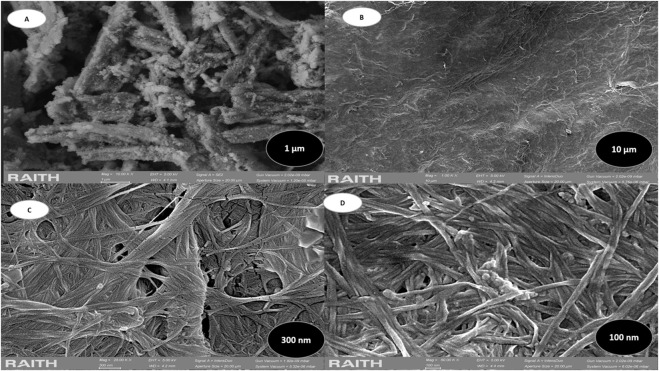
BC analysis. **(A)** Shows SEM morphology at 1 µm. **(B)** Shows SEM morphology at 10 µm. **(C)** Shows SEM morphology at 300 nm. **(D)** Shows SEM morphology at 100 nm.

Following the completion of six adsorption cycles, an isotherm evaluation process was initiated in order to determine the behaviours and evaluate the new forms. It is evident that bacterial cellulose, in conjunction with ferric chloride, exhibits a monolayer and a homogeneous surface as a consequence of its purity, with a cellulose component that is 100% pure. It is evident that the Langmuir isotherm is the most appropriate model to describe the system under investigation. However, following the adsorption and elution process with EDTA, a deviation from this model becomes apparent. This deviation is characterised by the presence of lumps and alterations in the homogeneous composition of the surface, which renders the Freundlich isotherm a more suitable model for description, with an R^2^ fit of 0.95. As illustrated in Figure 7, the characterisation after six treatment cycles.

**FIGURE 8:**
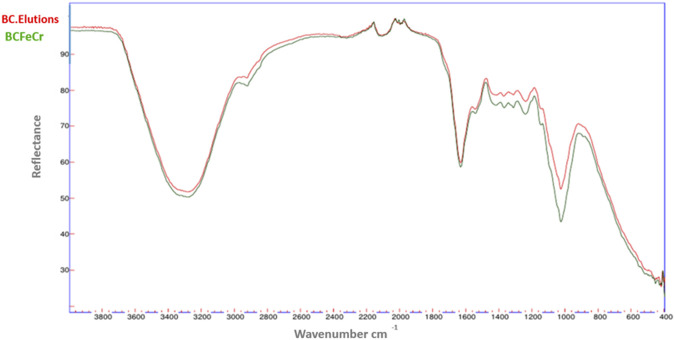
Analysed of FTIR before of cycle six.

A comparison of the images in Figure 7 (see Figures 7A–C) reveals some characteristic deterioration after elution and subsequent reuse. Porosities and poor homogeneity are observed at the nanofibre level, characteristics that disappear after the reuse and recycling process. The loss of homogeneity of BCFe (2) and the resultant wear are the primary causes of this decline. Consequently, the biomass transitions from a Langmuir to a Freundlich isotherm. As illustrated in Figure 8, an FTIR analysis is displayed for treatment cycle six.

As illustrated in Figure 8, a decline in the bacterial cellulose biomass was evident in the OH groups. However, subsequent to the elution processes, a recovery of this biomass was observed through elution with EDTA, thereby demonstrating a notable degree of resilience to the elution processes and subsequent reuse. The peaks (C=O and CO) also exhibited an increase following elutions with EDTA, which, as with the OH group, demonstrated recovery, albeit with reduced intensity.

## 4 Conclusion

Treatment systems utilizing bacterial cellulose and three concentrations of Ferric chloride were developed for the remediation of Cr (VI). The treatment system comprising 85% bacterial cellulose and 15% Ferric chloride was identified as the most effective due to its high adsorption capacity, which reached 456 mg/g after multiple reuse cycles. The biomass composition transitioned from the Langmuir isotherm to the Freundlich isotherm, indicating alterations to its biochemical structure. Following elution with EDTA, a decline in adsorption capacity was observed, suggesting a shift in the system’s equilibrium. The treatment system with these compositions is now ready to be deployed in industries that contaminate the environment with Cr (VI), and could be adapted to other contaminants, with ease of implementation and scalability.

## Data Availability

The raw data supporting the conclusions of this article will be made available by the authors, without undue reservation.
